# Equity and timeliness as factors in the effectiveness of an ethical prenatal sequencing service: reflections from parents and professionals

**DOI:** 10.1038/s41431-024-01700-0

**Published:** 2024-10-03

**Authors:** Michelle Peter, Melissa Hill, Jane Fisher, Morgan Daniel, Hannah McInnes-Dean, Rhiannon Mellis, Holly Walton, Caroline Lafarge, Kerry Leeson-Beevers, Sophie Peet, Dagmar Tapon, Sarah L. Wynn, Lyn S. Chitty, Michael Parker

**Affiliations:** 1 North Thames Genomic Laboratory Hub, Great Ormond Street Hospital for Children NHS Foundation Trust, London, UK; 2 Genetics and Genomic Medicine, UCL Great Ormond Street Institute of Child Health, London, UK; 3 Antenatal Results and Choices, London, UK; 4 Behavioural Science and Health, Institute of Epidemiology and Health Care, University College London, London, UK; 5 School of Human and Social Sciences, University of West London, London, UK; 6 Alström Syndrome UK, Torquay, UK; 7 Genetic Alliance UK, London, UK; 8 Queen Charlotte’s & Chelsea Hospital, Imperial College Healthcare NHS Trust, London, UK; 9 Unique - Rare Chromosome Disorder Support Group, Oxted, UK; 10 The Ethox Centre, Nuffield Department of Population Health and Wellcome Centre for Ethics and Humanities, University of Oxford, Oxford, UK

**Keywords:** Genetic testing, Genetic testing, Ethics, Next-generation sequencing

## Abstract

Prenatal sequencing tests are being introduced into clinical practice in many developed countries. In part due to its greater ability to detect genetic variation, offering prenatal sequencing can present ethical challenges. Here we review ethical issues arising following the implementation of prenatal sequencing in the English National Health Service (NHS). We analysed semi structured interviews conducted with 48 parents offered prenatal sequencing and 63 health professionals involved in delivering the service to identify the ethical issues raised. Two main themes were identified: (1) Equity of access (including issues around eligibility criteria, laboratory analytical processes, awareness and education of clinicians, fear of litigation, geography, parental travel costs, and access to private healthcare), and (2) Timeliness and its impact on parental decision-making in pregnancy (in the context of the law around termination of pregnancy, decision-making in the absence of prenatal sequencing results, and the “importance” of prenatal sequencing results). Recognising both the practical and systemic ethical issues that arise out of delivering a national prenatal sequencing service is crucial. Although specific to the English context, many of the issues we identified are applicable to prenatal sequencing services more broadly. Education of health professionals and parents will help to mitigate some of these ethical issues.

## Introduction

There is a growing literature on the ethical questions presented by the use of genomic sequencing tests in a prenatal setting, with a number of professional guidelines published on practical ethical issues and how to address them [1–9]. These practical ethical issues include the challenges of achieving valid consent, the management of patient expectations, the return of results, dealing with incidental findings, and implications of results for family members. Debates around the ethics of prenatal testing are long-standing whereby it has been positioned by some as a mechanism for reinforcing ableism [10–12], and others as a way to support reproductive choice [13]. There is also a discourse on the morality of prenatal testing as a way of selecting against disabling traits and avoiding the birth of children with certain conditions [14–16]. Thus, whilst the use of genomic technologies for prenatal diagnosis may be new, many of the ethical issues that arise from providing parents with information in the prenatal period are not.

As genomic sequencing continues to be adopted into clinical practice more widely, ethical issues will be an important consideration for optimal implementation. Reflecting on the ethical complexities of prenatal genomics is particularly timely in the English healthcare context where prenatal exome sequencing (pES) was made available in October 2020 as a national service delivered through the National Health Service (NHS) Genomic Medicine Service (GMS). The EXPRESS study is a multidisciplinary evaluation of the national implementation of the NHS prenatal exome sequencing (pES) service [17]. As part of this study here, we examine the ethical issues arising from the introduction of pES using qualitative interviews with parents offered pES and health professionals involved in delivering the service.

Interview participants in this study raised many of the practical ethical considerations outlined above that have been previously described in the literature [1–9]. They also highlighted structural and systemic ethical issues which, as far as we are aware, have not been explored in previous empirical work. For this reason, this manuscript focuses on these ethical issues, gathering them under two interrelated and, to some degree, interdependent sets of issues: those concerning questions of equity, and those relating to the challenges for decision making that arise out of the time constraints created by the intersection of legal, health system, and biological timelines.

## Subjects and methods

### Study design

This was a nationwide study investigating the ethical issues impacting the provision of pES in England. The study draws on qualitative interviews conducted with professionals involved in delivering pES, and parents who have been offered pES. Detailed methods and the findings relating to professional [18] and parent experiences [19] have been reported elsewhere. Our approach for this work specifically was to identify within the interviews key issues with an ethical focus. Using moral imagination to consider to what extent core principles of prenatal diagnostic testing are consistent (or inconsistent) with the ethical trade-offs, we focused on issues of fairness and justice with respect to moral and professional codes of ethics.

### Setting

pES is offered nationally in England through the NHS GMS. pES is offered in pregnancies where structural anomalies are thought likely to have a genetic aetiology, as determined by a multidisciplinary team that includes fetal medicine and genetics experts [20]. Ideally whole exome sequencing is performed on the trio (fetus, biological mother and father), with subsequent analysis performed using a panel of more than 1300 genes known to cause structural anomalies detectable by imaging in the prenatal or early neonatal period [21]. Variants of uncertain significance (VUS) are reported in some circumstances following multidisciplinary team review. Incidental findings with implications for child or parental health, or future reproductive risks are reported. Additional findings, such as cancer susceptibility genes, are not looked for. Although the rapid testing pathway is aimed primarily at those for whom a result may influence future pregnancies, a non-urgent exome sequencing service is available for foetal testing where termination of pregnancy has already been decided or when foetal demise has occurred or is imminent. The target turnaround time for a result in the non-urgent pathway is within 84 days from receipt of all necessary samples and paperwork.

### Recruitment

Professionals, including clinical geneticists, fetal medicine specialists, midwives, genetic counsellors, and laboratory scientists from across England involved in delivering pES were sent a study invitation and participant information. Parents over 18 years who had been offered pES in the NHS GMS were recruited through the parent support charity Antenatal Results and Choices (ARC) and Fetal Medicine Units (FMUs) at six NHS hospitals. A study invitation aimed at parents was posted on the ARC website or sent by mail with a follow-up phone call from the clinical team through the potential participant’s FMU. Parents who took part in an interview were offered a £10 gift voucher as reimbursement for their time. Written or audio-recorded verbal consent was obtained from each participant prior to the interview.

### Interviews

Sixty-three interviews with professionals and 42 interviews with parents (six with male partners present) were conducted. Interview topic guides explored parent experiences with pES, parent information and support needs pre- and post-pES, benefits and concerns around pES (including ethical concerns), and impact of pES results on decisions regarding pregnancy management (see Topic guides in Supplementary Materials). Professionals were asked about the structure and delivery of the pES service. Standard demographic questions were included (Table 1). Interviews were audio-recorded, transcribed verbatim, and pseudo-anonymised prior to analysis.

**Table 1:** Participant characteristics (adapted from McInnes-Dean et al., 2024).

	*N*	(%)		*N*	(%)
*Parent participants*			*Professional participants*		
**Gender**			**Professional role**	24	38%
Female	42	88%	Clinical genetics clinician	21	33%
Male	6	13%	Fetal medicine clinician	6	10%
**Ethnicity**			Fetal medicine midwife	7	11%
White/White British	39	81%	Genetic counsellor	5	8%
Asian/Asian British	4	8%	Clinical scientist		
Other	2	4%	**Region in England**		
Black/Black British	2	4%	North West GMSA	8	13%
Mixed	1	2%	North East and Yorkshire GMSA	8	13%
**Education**			East GMSA	10	16%
Degree or above	37	77%	Central and South GMSA	11	17%
Vocational	5	10%	North Thames GMSA	12	19%
GCSE/O-level	4	8%	South East GMSA	4	6%
A-level	1	2%	South West GMSA	7	11%
No qualification	0	0%	NA	3	5%
Unknown	1	2%			
**Main language spoken**					
English	36	75%			
Other	4	8%			
Unknown	8	17%			
**Religion**					
Christian	12	25%			
None	25	52%			
Sikh	1	2%			
Agnostic	1	2%			
Unknown	9	19%			
**Age (years)**	34.5				
Mean	35				
Median	(28–49)				
Range					

The above numbers reflect the inclusion of 48 parent participants; only 42 parent interviews were conducted since six interviews included a couple.

*Key*: GMSA = National Health Service Genomic Medicine Service Alliance (there are seven GMSAs that coordinate the embedding of genomics into mainstream clinical care in England).

### Data analysis

Analysis of interviews was facilitated by NVivo version 13 (QSR International, Pty Ltd) and followed the principles of thematic analysis [22] with findings generated using a team-based codebook approach [23] that combined inductive and deductive approaches [24]. Further details regarding our primary analysis have been published elsewhere [18, 19]. For the purpose of this paper, MParker and MPeter conducted a secondary analysis of the coded data through an ethical lens to draw out the ethical issues linked with implementing the pES service.

## Results

Interviews were conducted with 48 parents (42 pregnancies). pES was accepted in 40 pregnancies and declined in two. Where pES was accepted, 16 received a diagnosis or partial diagnosis, one received a VUS, and 23 had a ‘no findings’ result. Of all pregnancies, 21 chose termination. Participant characteristics for parents and professionals are presented in Table 1.

### Interview findings

**Fig. 1:**
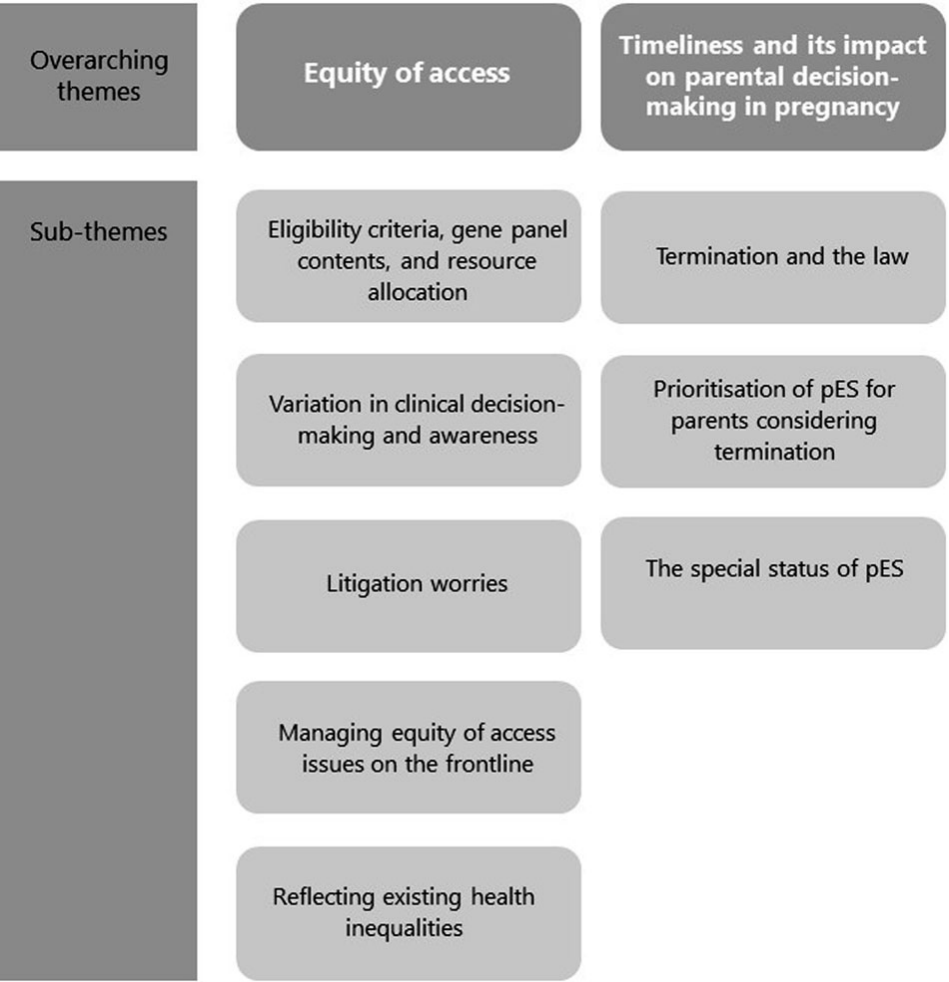
Themes and sub-themes from interviews with parents and professionals.

Our findings are described within two overarching themes: ‘Equity of access’ and ‘Timeliness and its impact on parental decision-making in pregnancy’ (Fig. 1).

### Equity of access

#### Eligibility criteria, gene panel contents, and resource allocation

Issues around equity of access to pES were mentioned by both parents and professionals - for example in the context of the pES eligibility criteria, which had been informed by published literature and developed through consultation with clinicians. There were clear differences of opinion amongst professionals about whether these criteria were appropriate, with some considering them ‘sensible’ and ‘a good starting point’, and others viewing them as too narrow and limiting parental access to pES (Table 2, Q1). Some parents also supported wider criteria and wanted to make the service available to all who might find it useful (Table 2, Q2).

**Table 2:** Equity of access.

**Eligibility criteria, gene panel contents, and resource allocation**	
Q1	“It will be good when we can broaden the inclusion criteria because I suspect that actually there are cases out there that are not included in the check list that we’re missing. And I think that’s important from a parent’s perspective.” Professional 2 — FMU Consultant.
Q2	“So, yeah, we’re really fortunate that the counsellor pushed and said ‘no please go ahead and do it anyway’, so I’d maybe consider what the criteria are and whether it could be more broad in those kind of circumstances, because we could quite easily have not found these results out.” Parents 7 & 8, diagnosis from pES (termination of pregnancy).
Q3	“We’re hitting a diagnostic yield of 40% which is excellent when you look at a test and what a high diagnostic yield, but we must be missing some and those cases are going to come back to bite us at some point potentially.” Professional 60 — Clinical geneticist.
Q4	“Well, financially, absolutely it is appropriate because money matters and if one hasn’t got enough money and resources to do a test for everyone then one has to ration it. Distributive justice has always existed in healthcare…I aspire that there would be the ability in the future to offer the test to many more people, but currently I understand that we are bound by rules of efficiency and rules of cost efficiency.” Professional 14—FMU Consultant.
Q5	“I understand there’s obviously a big cost with it and all of that, but the more research and development that can go into making that nice and clear so that people can access it when appropriate the better really, because it made all the difference for us I would say.” Parents 31 & 32, diagnosis from pES (termination of pregnancy).
**Variation in clinical decision-making and awareness**	
Q6	“I don’t think it’s standardised anyway. You will never remove a degree of individuality from discussions of any nature, whether you’ve got eligibility criteria or not because there’s always a degree of human interpretation, particularly when you’re thinking about things that present prenatally.” Professional 59 - Clinical geneticist.
Q7	“I mean a classic is…’I think this is Down’s syndrome, so we’ll do a QF-PCR’. ‘Oh, it’s not Down’s syndrome. Oh, we’ll refer you to [hospital 1]’. By which time you’ve gone on so many weeks and they come and see you…and they’ve obviously missed the care that they might have received had they come straight to [hospital 1].” Professional 36 – FMU consultant.
Q8	“There’s lots of peripheral hospitals that don’t always refer to our FMU, so if they don’t get referred to our FMU then they don’t get access to this service…I think some of it’s about cost because they get charged for tertiary referrals…I think some of it is they feel like they can manage it and they just think well what else can they offer or they feel like they can do it all.” Professional 60 - Clinical geneticist.
Q9	“I know other units have said everyone comes to the tertiary centre, but particularly in [place] where people don’t want to travel, don’t have the money to travel, can’t travel, they will be very insistent on staying local for as long as they can…Whereas if they were local to [hospital] in the same socioeconomic class, they would have received that.” Professional 36 – FMU Consultant.
Q10	“I remember going there, having done like an NHS appointment a few days prior and them telling us, like, we’ve ruled out everything severe that we think and we think you’re fine…and then like ‘no actually there could still be something seriously wrong’ and that they would have just let us continue with it if it really wasn’t for that private doctor pushing for us to ask for exome sequencing.” Parent 29 – mother, diagnosis from pES (termination of pregnancy).
**Litigation worries**	
Q11	“What happens if I say this is monogenic, […] refuse [pES] and then they have a child with Kabuki Syndrome—what happens then?” Professional 53 — Clinical geneticist.
Q12	“I’d say that in fetal medicine, in general, there is a big concern about wrongful birth…And so there is a real push to want to use this as a test of exclusion. If I haven’t offered that test may I then be told that I should have offered that test?.” Professional 59 — Clinical geneticist.
**Managing equity of access issues on the frontline**	
Q13	“Getting parents and the baby DNA is difficult, particularly dads and the delay in the geneticist approval means that you don’t know sometimes whether you should take the consent and do the bloods because you may not have another opportunity to but then you’re setting up the expectations of the parents unrealistically or unfairly.” Professional 3 – FMU Consultant.
Q14	“The patients are starting to find out now, which is good, but that puts more pressure on because you have a couple who are educated, they know what’s available, they want the test, there’s a good reason for doing it and then the lab refuse to do it, I mean, what position does that put us in?” Professional 53 - Clinical geneticist.
**Reflecting existing health inequalities**	
Q15	“So we travelled by car, that was absolutely fine for us, no problem…if you didn’t have a car that would have been a big overhead, public transport and all that stuff…we didn’t have to worry about it, but I guess that is a consideration for patients who can’t do that.” Parents 7 & 8, diagnosis from pES, (termination of pregnancy).
Q16	“I had a lady last week to consent and…I was lucky because the mum understood me but couldn’t really speak very good English. The interpreter started and the mum interrupted and she said, “She’s not saying the same as you” and then the interpreter put the phone down because basically she just said “The baby’s heart’s abnormal” and what I was saying to her was in much more detail than that.” Professional 34 — FMU consultant,

Professionals described the ethical challenge of testing for some conditions and not others. When interviews were conducted, genomic analysis for pES was based on a panel of around 1200 genes where variants are known to cause fetal or neonatal structural anomalies. This approach to analysis was questioned by some professionals who felt the diagnostic yield for pES (33.6%, North Thames GLH and Central and South GLH, May 2024) was ‘too high’ and that the test is ‘very selective’ in the conditions it can identify (Table 2, Q3). Many professionals did, however, recognise a dilemma associated with a broader panel approach, noting that, while testing for a wider range of conditions could provide access to pES for more parents, ‘increasing the fishing net’ would also increase the risk of identifying VUSs, thus creating uncertainty for parents.

Professionals also acknowledged that difficult resource allocation decisions were an inevitable part of publicly funded health services (Table 2, Q4). Notably, balancing cost against societal value was also recognised by parents who highlighted a need for clear, equitable processes for priority setting in the face of limited resources (Table 2, Q5).

#### Variation in clinical decision-making and awareness

Implications for equity also arise out of the individual judgements of clinicians, with views that eligibility decisions are sometimes subject to individual interpretation (Table 2, Q6), or differences in clinicians’ awareness of pES or their understanding of the eligibility criteria and referral processes. These knowledge gaps can potentially lead to delays for parents in accessing pES through a tertiary centre (Table 2, Q7). In addition, there was evidence that local decisions about pES referrals were impacted by resource issues, highlighting the interaction between individual or institutional referral practices and financial considerations (Table 2, Q8).

Some professionals highlighted differential access to ‘proper foetal medicine phenotyping’ (Professional 37 – FMU Consultant) as another way in which the realities of practice introduce inequity. Where expert foetal imaging is unavailable locally, parents may be required to travel for specialist care and must balance the feasibility and practicalities of attending their clinic appointment against their desire for information from pES. The costs of travel and childcare could also act as barriers to access (Table 2, Q9). Variation in the level of specialist input at multidisciplinary team (MDT) meetings was also felt to contribute to inequity, with MDTs in some hospitals attended by prenatal geneticists whilst others operated without regular access to this expertise.

Equity issues also include parents’ ability to access private healthcare, with some parents seeking additional opinions in the private sector which resulted in them gaining access to pES (Table 2, Q10). Navigating these situations was clearly difficult for parents, but these scenarios also present a complex moral and ethical challenge for clinicians who may feel undue pressure to offer pES when they do not feel it is necessary.

#### Litigation worries

Some professionals felt that referral for pES could be influenced by worries about the implications of not offering it. Many were concerned that, in our ‘increasingly litigious and entitled society’ (Professional 1 — Clinical geneticist), clinicians could be sued if pES was not offered or refused, and a genetic condition discovered after birth (Table 2, Q11). Professionals also noted that these worries could influence referral practice in ways that stretched the application of eligibility criteria (Table 2, Q12), resulting in significant variation in parental access to pES.

#### Managing equity of access issues on the frontline

Participants described the ethical complexities of managing parents’ expectations around access to pES, particularly when there were uncertainties around whether the case would meet the eligibility criteria. Similarly, care pathways that minimise delays by opportunistically collecting the blood samples for pES before eligibility is confirmed, require clinicians to be careful not to build false hope that the referral will be accepted (Table 2, Q13). Professionals also noted that an increased public awareness of pES places pressure on professionals to carefully manage parents’ expectations of being offered testing as the eligibility criteria may not be met (Table 2, Q14). Notably, several parents described the stress and frustration of waiting for eligibility to be confirmed when it was unclear whether their pregnancy would meet the criteria.

#### Reflecting existing health inequalities

Though not unique to pES, social and health inequalities were seen to impact access as parents constrained by geographic and financial boundaries were felt to be less likely to benefit. One professional described an example of pES results being delayed because the parents could not afford to travel to have a blood sample taken. Notably, the costs associated with travel had not prevented any of the parents we interviewed from attending their appointments; the need for information outweighed the financial costs incurred. However, there was acknowledgement that travel costs are not affordable to everyone (Table 2, Q15) and that families with different financial priorities may not be in a position to choose.

Whilst not confined to the pES setting, discussing nuanced and complex information with parents with learning disabilities, limited education or whose first language is not English was described by professionals as difficult and could bring about inequity. Experiences with interpreter services were mixed; it could sometimes be challenging to book an interpreter at the required time and while many individual interpreters were very good, some conveyed limited or inaccurate information (Table 2, Q16) which can lead to misunderstandings that impact informed decision-making.

### Timeliness and its impact on parental decision-making in pregnancy

#### Termination and the law

The ethical issues that parents and professionals found most challenging were those arising out of the interrelationships between the timelines linked to pES, the requirements of the laws around termination, and the decision-making needs of parents. Timeliness of referral and return of pES results can be crucial for parents wanting to use this information to guide decisions about continuing or ending their pregnancy. It is, however, important to note that these decisions are not always contingent on pES results; in some cases, decisions can be based on scan findings that show definitively that the fetus is severely affected.

In our interviews, parents and professionals described the challenges of the time sensitive nature of pES for parental decision-making around termination of pregnancy (Table 3, Q1). These challenges arise as most referrals for pES are made following the identification of fetal structural anomalies at the routine 18–20-week fetal anomaly scan. This is close to 24 weeks where the law in England changes and termination is only permitted if there is ‘substantial risk’ of serious impairment in the child if born. In addition, the timelines for pES can be impacted by multiple factors, such as delays in obtaining parent samples, additional investigations prior pES referral or the need for further investigations to clarify a potential diagnosis following whole exome sequencing (Table 3, Q2).

**Table 3:** Timeliness and its impact on parental decision-making in pregnancy.

**Termination and the law**	
Q1	“A lot of the information is only becoming available after 24 weeks…if they pin their hopes on getting a diagnosis to be able to justify the option of a termination and that result comes through after 24 weeks, it’s quite possible that with the absence of a genetic diagnosis the option of termination is no longer available.” Professional 7 — FMU consultant.
Q2	“If we find an anomaly at twenty weeks and then we start doing foetal echoes and MRI scans, and then we decide to do an exome, they’re after twenty-four weeks by the time we get the result back or just before, and they’re making rush decisions.” Professional 60 — Clinical geneticist.
Q3	“Time was against us so if there was an issue, we didn’t want this pregnancy to go on much longer, you know, naturally 24 weeks you’re very far down the line by this point so I think we had to act quite quickly to think what was the best thing to do.” Parent 40 — mother, no findings result from pES (live pregnancy).
**Prioritisation of pES for parents considering termination**	
Q4	“So anyway, ten to fourteen days for the results… by the end of the fourteen days we still didn’t know anything, we hadn’t heard anything back…we kind of were at the deadline of having to make our decision. So, my hospital got us in for a meeting, explained the statistics, they put a plan for the process of termination and then I think, even by the time of the termination, we still didn’t have the exome sequencing results.” Parent 1- mother, partial diagnosis from pES (termination of pregnancy)
Q5	“…obviously one of the main criteria for having an exome is that it would alter the management of the pregnancy in some way and [the local FMU clinicians] don’t get that…so we’ve got one ongoing at the moment that has got catastrophic brain findings, absolutely, there’s absolutely no way this is going to be a normal functioning child. The parents will not terminate, don’t want to terminate, want an exome, I said ‘well what’s the point…what is the purpose of this very expensive test if it’s not going to change the management… there is limited capacity and it’s a very explicit requirement, it must have the potential to change the management of the pregnancy”. Professional – 46, Clinical geneticist.
**The special status of pES**	
Q6	“That was one of my worst fears, that we would have no answer [from pES] and then they would be asking us our decision on whether we were going to terminate or not based on no hard facts”. Parent 2 — mother, no findings from pES (termination of pregnancy).
Q7	“So, we definitely notice that people’s understanding of that test is better news to not find anything, which may be true but isn’t always true. And so I think the problem is, when people understand a little bit…you’re spending more time unpicking what they actually understand already…and unpicking all of that is actually sometimes harder than starting from nothing.” Professional 52 - Genetic counsellor.

From a parent perspective, the intersecting timelines of the pES testing process and England’s 24-week termination limit was described as ‘incredibly tight’ and ‘stressful’ (Table 3, Q3). Worries about timing were a source of great stress and anxiety noted by both parents and professionals, with parents feeling they have to make rapid, difficult, life-changing decisions. Ultimately the interconnectedness of legal, technological and health system timelines meant that some parents had to make decisions about their pregnancy prior to the return of pES results (Table 3, Q4).

#### Prioritisation of pES for parents considering termination

Some parents were made to feel that access to pES was dependent on whether or not they planned to continue their pregnancy. For example, one parent described being asked on multiple occasions whether she planned to continue her pregnancy because if so, the test would not be run. She also recalled feeling added pressure to confirm her stance on termination of her pregnancy after a clinician mentioned the cost of the test, stating, “that’s what put me under pressure, and I didn’t like it at all” (Parent 28 — mother, diagnosis from pES (termination of pregnancy)).

From a parent perspective, prioritisation of pES for those whose termination decision will be informed by the result could be experienced as distressing and unjust. However, from the perspective of a health system that needs to consider the needs of all parents, the prioritisation of those whose decision hangs on the test result makes financial sense (Table 3, Q5).

#### The special status of pES

It was clear that pES can be seen by parents and professionals as the most important—and sometimes overriding—component in the assessment of risk and in pregnancy management. Although pES is just one source of information amongst others, such as scan results, pES could be viewed as key for decision-making and the relevance of other results overlooked (Table 3, Q6).

The importance of training for clinicians to be able to interpret a ‘no findings’ result from pES in the context of other clinical findings and convey this information to parents was highlighted. Understanding the nuances of a ‘no findings’ result was also true for parents. Some professionals were concerned that parents could be overly reassured by a ‘no findings’ result. Careful counselling is needed to ensure that parents understand that this result does not eliminate the possibility of the baby having a genetic condition (Table 3, Q7), and that the anomalies seen on scan have implications for prognosis.

## Discussion

The analysis presented here illustrates the value of undertaking an integrated ‘equity and ethics impact assessment’ as part of the evaluation of health systems interventions and its potential to inform the provision of better and more equitable services for patients and families. Here, we explored the experiences of health professionals and parents through an ethical lens and identified two clusters of structural ethical factors that are important for the successful and equitable provision of pES.

The first cluster of structural issues comprised a range of interacting practical ways in which equity of access to pES can be threatened. Decisions on eligibility criteria and gene panel content were common issues that were linked by participants to equity, as they govern access to the test and the conditions tested for. Many participants, however, also recognised that healthcare resources are limited and that difficult prioritising decisions are needed that may restrict access to pES. It was also apparent that factors including variation in clinician referral practices, lack of awareness at peripheral units of the availability of pES services, and concerns about litigation impacted on equity of access. Many of these issues can be addressed by improving awareness through education of all stakeholders.

Our second cluster of structural ethical issues were linked to the timeliness of pES and its impact on decisions to continue or terminate the pregnancy. Many of these issues hinge on the change in the legal basis for termination of pregnancy that occurs at 24 weeks gestation whereby termination is only permitted if “there is a substantial risk that if the child were born it would suffer from such physical or mental abnormality as to be seriously handicapped”. For our participants, the change in law at 24 weeks meant that timely referral and the efficient return of results to parents were essential requirements for an effective and ethical pES service. Waiting for the results of pES when close to 24 weeks was an extremely stressful experience for many parents and for the clinicians who care for them. Indeed, some parents felt they could not wait for pES results as the deadline drew near and made decisions about ending or continuing the pregnancy in the absence of the additional information from pES. The relationship between pES and termination of pregnancy is complex and multifaceted, and likely to change over time. Whilst there are some cases in which a pES finding informs a decision to terminate, there are others in which results show a less severe condition than expected allowing parents to decide to continue the pregnancy. In the future, as therapies become available it is possible that pES will come to play a role in identifying opportunities for early intervention.

Our analysis identified that the two clusters of ethically relevant problems—issues around equity of access and those relating to timeliness – have the potential to be mutually reinforcing. That is, problems arising out of inequity of access to services and the nature of referrals for pES based on findings from the 20-week fetal anomaly scan (or later if additional tests such as MRI are required) could make it harder to achieve timely return of results. This has the inevitable consequence that some parents’ decisions about termination of pregnancy will be made later in pregnancy or potentially without information from pES. It also seems likely that the resulting time constraints on decision-making will in turn impact those who are already disadvantaged in other ways. This suggests that there are strong equity-based reasons for paying close attention to addressing both the structural drivers of inequity and looking for ways to improve the timeliness of both referral processes and the processes of pES themselves. However, it is important to acknowledge that improving timelines is challenging as many anomalies are not detected until the routine fetal anomaly scan at 18–20 weeks. It has been suggested that the number of late terminations following pES testing could be reduced by the introduction of an earlier assessment of fetal anatomy since this would allow earlier identification of some of the cases eligible for pES [25], but this would not address the issue entirely as some fetal anomalies do not present until late in pregnancy. Fetal anomaly screening is, however, continuing to evolve with new technologies becoming available that may improve detection and impact timelines, at least in some cases [26].

A key finding from this analysis was that some parents and professionals placed special and sometimes overriding importance on pES, despite the availability of other relevant information such as a prenatal ultrasound scan or MRI findings. For example, a fetus with a very small chest and short limbs has a high chance of an underlying lethal abnormality regardless of the pES result, while a fetus with severe brain anomalies is likely to have significant developmental delay. In these circumstances counselling can be based on imaging findings, and although genetic results can influence later reproductive decision making, these are not crucial for managing the current pregnancy. Information from scans and other sources is important when interpreting a ‘no findings’ pES result, especially as professionals raised concerns that parents could be overly reassured by this result. Furthermore, other studies have shown that parents can adopt both pessimistic and optimistic interpretations to no informative findings from pES [27].

Whilst our interpretation is specific to the offer of pES in England, we anticipate that the ethical challenges we have described will be experienced in other contexts as pES is adopted more widely. Regardless of the health system in which pES is offered, clinicians must be aware of the limitations of pES and the need to interpret results in the context of other available information in order to deliver personalised post-test counselling and support for parents. Furthermore, all health systems in which pES is offered should prioritise education and training for clinicians on the clinical indications for pES. This is fundamental for supporting clinician confidence and avoiding defensive medical practice. Finally, issues around equitable access are relevant to all healthcare systems, but are especially pronounced for those in which pES is available only via out of pocket costs or specific insurance coverage. Careful attention must be paid to avoid exacerbating health inequities and to ensure that all parents who may benefit from pES are able to access it.

### Limitations

A key limitation of our study is that the majority of our parent participants reported being White/White British and were educated to degree level or above. The majority had also opted to have pES. As a result, our participants do not represent the views and experiences of all parents offered pES. Further, there is a risk of responder bias since participants were self-selected.

## Conclusion

In this paper, we have identified several ways in which the identification and analysis of ethical questions can be useful in the evaluation of a new clinical service. Addressing the practical ethical questions arising for health professionals and parents such as those relating to consent, feedback of findings, implications for family members, and the scope of health professionals’ responsibilities is vital for its success and can, to some extent, be mitigated by improving stakeholder education. However, identification and addressing of structural and systematic ethical concerns also require careful attention. In our analysis presented here, these structural ethical issues are experienced by both health professionals and parents particularly in relation to questions of equity and of timeliness.

## Supplementary information

Topic guides for parents and professionals

## Data Availability

The raw qualitative data that support the findings of this study contain sensitive information and so we are unable to share the full transcripts from the interviews. However, we are able to provide a summary of interviews to other researchers upon reasonable request.
