# Supplementary material for: Equity and timeliness as factors in the effectiveness of an ethical prenatal sequencing service: reflections from parents and professionals
Source: Eur J Hum Genet. 2024 Oct 3;33(3):360–7. doi: 10.1038/s41431-024-01700-0 (PMC11894143; doi:10.1038/s41431-024-01700-0)
Supplement: Supplementary file 1 — Topic guides for parents and professionals [file 41431_2024_1700_MOESM1_ESM.pdf]

Interview Topic Guide – parents who accepted pES

Introduction

- Thank participant for taking part in the interview
- Explain that reason for this research is to understand people's views of rapid exome sequencing (ES) in pregnancy and consider what information and support parents need
- Recognise that this is a very sensitive topic and we can stop the interview at any time if it gets upsetting
- The participant can also choose to withdraw from study and delete the audio-recording at any time

Background

1. Demographic questions (age, ethnicity, education, religious affiliation, number of children)

Experiences of pregnancy and prenatal testing

2. Could you tell me about your pregnancy when you had a problem suspected following ultrasound?

3. Can you tell me about any options that were discussed with you about finding a diagnosis for the baby during your pregnancy? [prompt: different types of tests / additional scans]

4. Can you tell me what you remember about being offered ES?
   a. Who approached you? Prompt: was it a joint consultation – genetics/FMU
   b. Do you recall what was discussed?
   c. Had you heard of genome or exome sequencing before?
   d. What were you told about the results you might receive?
   e. Where any limitations of the test discussed? (prompt: such as genetic condition present that might not get picked up)
   f. Do you recall what you were told about why you and your partner also needed a test?
   g. What questions did you have at the time?
   h. Were you given any written/printed information?
   i. Did you feel you had a good understanding of ES from the information provided (written and verbal)?

5. What were the main reasons that led you to say yes to having ES?
   a. Who did you talk to when making this decision? (partner / wider family / health professionals)
   b. How did your partner feel about ES?
   c. How much time did you have to make the decision? Was this enough time?
   d. Did you weigh-up any concerns? (miscarriage risk, data security, data sharing, insurance, timing of results and decisions about termination)
   e. Given all that was going on at this time, what was it like to be having to make a decision about ES?
   f. What do you think influenced the decisions you made regarding prenatal testing? e.g. culture, ethnicity, religion, etc

6. What were your expectations of having rapid ES?
   a. Were you expecting to receive a diagnosis?

b. Had you considered that you might not get a result or that you might get an uncertain result?
c. Had you considered that you might find out something about yourself?

7. Can you tell me about receiving the results?
   a. How long did it take to get the results?
   b. When in your pregnancy did you receive the result?
   c. Do you recall who gave you the result?
      Prompt: was it a joint consultation – genetics/FMU
   d. What did the result mean for you?
   e. Was there anything uncertain about the result?

8. Did having ES influence your decision to continue/not continue the pregnancy?
   a. What factors influenced your decision?
   b. Who did you talk to when making this decision? (partner/family / professionals)

9. Were there particular times during the process of testing that you feel you (or your partner) would have benefited from more support or more information? (prompt: deciding about the test, waiting for results, deciding about termination of pregnancy)

10. Thinking back to the information you were given before and after testing; do you have any suggestions for how the information about ES could be improved?
    a. Were there any aspects of the discussion that were confusing or unclear?
    b. What additional information would you have liked at that time?

11. What do you think we need to think about when developing approaches to counselling and patient information sheets?
    a. What are the important topics to include?
    b. Do you think we need a lot of detail about how the ES test works?
    c. What format would you like the patient information to be in; eg written information sheets, interactive online information?
    d. What other support or signposting do you think you would need?
    e. Who do you think should offer this test? Should it be a professional you have come to know during your pregnancy such as a midwife or would you rather speak to a specialist in genetics or fetal medicine?

12. As part of our research, we would like to consider the financial costs of prenatal ES for parents
    a. When you were offered ES and went through the consent form do you recall if this was an in person appointment or a video consultation?
    b. Do you recall how long that appointment was?
    c. When you were told your ES results – how was this done? Letter, in person appointment or video consultation?
    d. Do you recall how long that appointment was?
    e. Did you have any additional appointments related to ES?
    f. [as appropriate] When you had these appointments did you need to arrange childcare?
       Prompt: If yes, did you pay someone to look after them? Did someone have to take time off work to look after them?
    g. [as appropriate] Did you have to take time off work for the appointments?

Prompt: If yes, was this time off: Paid absence from work? Unpaid absence from work? Will make the time up? Came to clinic outside work time? Took holiday? Other arrangements?

h. [if there is time to ask this] Do you recall how you travelled to your appointment(s)? Prompt: If you came by private car, can you remember how much was paid in car park fees? If by public transport, can you remember how much it cost?

i. Other costs? Prompt: extra appointments, travel / other costs

13. What are your overall thoughts about prenatal ES?
    a. What do you think are the main benefits and disadvantages?
    b. Do you have any concerns about it being offered more widely?

Issues others have raised

14. Sometimes with these sorts of tests that look at large amounts of your genetic information, there can be findings that are sometimes called incidental, additional or secondary findings. This is when the scientists doing the test find unexpected gene changes not related to the suspected condition in the baby. These findings could impact on health, such as gene changes that increase your risk of cancer or heart disease.

There is always a very small chance that incidental or additional findings might come up. However, it is also possible to have a service where parents can choose to have scientists look for additional findings for themselves or their baby. **This is not the case for the NHS service at present.**

a. What do you think of offering incidental findings alongside ES in pregnancy – for yourself / partner / baby?
b. What do you think are the benefits / what would be your concerns?

15. At present rapid prenatal ES requires an invasive test, which has a risk of miscarriage. What do you think about offering ES as a non-invasive maternal blood test?
    a. Is it a good thing / bad thing?
    b. Are there additional issues to consider for information and counselling?

Interview Topic Guide – parents who declined pES

Introduction

- Thank the participant for taking part in the interview
- Explain that reason for this research is to understand people's views of rapid exome sequencing (ES) in pregnancy and consider what information and support parents need
- Recognise that this is a very sensitive topic and we can stop the interview at any time if they find it upsetting
- The participant can also choose to withdraw from study and delete the audio-recording at any time

Background

1. Demographic questions (age, ethnicity, education, religious affiliation, number of children)

Experiences of pregnancy and prenatal testing

2. Could you tell me about your pregnancy when you had a problem suspected following ultrasound?

3. Can you tell me about any options that were discussed with you about finding a diagnosis at this time? (Prompt: different types of tests / additional scans)

4. Can you tell me what you remember about being offered ES?
    a. Who approached you?
    b. Do you recall what was discussed?
    c. Had you heard of genome or exome sequencing before?
    d. What were you told about the results you might receive?
    e. Where any limitations of the test discussed? (prompt: such as genetic condition present that might not get picked up)
    f. Do you recall what you were told about why you and your partner also needed a test?
    g. What questions did you have at the time?
    h. Were you given any written/printed information?
    i. Did you feel you had a good understanding of ES from the information provided (written and verbal)?

5. What were the main reasons that led you to say no to ES?
    a. Did you have any concerns about ES? (miscarriage, data security, data sharing, insurance)
    b. Who did you talk to when making this decision? (partner / wider family / health professionals)
    c. How did your partner feel about being offered ES?
    d. How much time did you have to make the decision? Was this enough time?
    e. Given all that was going on at this time, what was it like to be having to make a decision about ES?
    f. What do you think influenced the decisions you made regarding prenatal testing? e.g. culture, ethnicity, religion, etc

6. Can you tell me about how you made your decision to continue/not continue the pregnancy?
    a. What factors influenced your decision?

    b. Who did you talk to when making this decision? (partner / family / other health professionals)

7. Were there particular times during the process of testing that you feel you would have benefited from more support or more information? (prompt: deciding about the test, deciding about termination of pregnancy)

8. Thinking back to the information you were given when offered ES; do you have any suggestions for how the information about ES could be improved?
    a. Were there any aspects of the discussion that were confusing or unclear?
    b. What additional information would you have liked at that time?

9. What do you think we need to think about when developing approaches to counselling and patient information sheets?
    a. What are the important topics to include?
    b. Do you think it is important to give a lot of detail about how the ES test works?
    c. What format would you like the patient information to be in; eg written information sheets, interactive online information?
    d. What other support or signposting do you think you would need?
    e. Who do you think should offer this test? Should it be a professional you have come to know during your pregnancy such as a midwife or would you rather speak to a specialist in genetics or fetal medicine?

General views on offering prenatal ES

10. What are your overall thoughts about prenatal ES?
    a. What do you think are the main benefits and disadvantages?
    b. Do you have any concerns about it being offered more widely on the NHS?

Issues others have raised

11. Sometimes with these sorts of tests that look at large amounts of your genetic information, there can be findings that are sometimes called incidental, additional or secondary findings. This is when the scientists doing the test find unexpected gene changes not related to the suspected condition in the baby. These findings could impact on health, such as gene changes that increase your risk of cancer or heart disease.

    There is always a very small chance that incidental or additional findings might come up. However, it is also possible to have a service where parents can choose to have the scientists look for additional health-related findings for themselves or their baby. **This is not the case for the NHS service at present.**

    a. What do you think of offering incidental findings alongside ES in pregnancy – for yourself / partner / baby?
    b. What do you think are the benefits / what would be your concerns?

12. At present rapid prenatal ES requires an invasive test, which has a risk of miscarriage. What do you think about offering ES as a non-invasive maternal blood test?
    a. Is it a good thing / bad thing?
    b. Are there additional issues to consider for information and counselling?

Interview Topic Guide

Professionals involved in delivering the rapid prenatal exome sequencing service at each GLH

Introduction

- Thank participant for taking part in the interview
- Explain that reason for this research is to understand how the service has been set up in their GLH and the challenges and successes
- Taking part is voluntary. The participant can also choose to withdraw from study and delete the audio-recording at any time.

Background

1. What is your professional background?

2. Can you talk to me a little about your current role and how long you have been in this position?

Views on the GMS

3. What are your thoughts on the new Genomic Medicine Service and the plan to embed genomic medicine in the NHS?
    a. What do you think about the reconfiguration of the genetic services into hubs?

4. Thinking about the GMS as a whole, what do you see as the main opportunities and benefits the GMS will provide now and in the future?

5. What do you anticipate the main challenges to incorporating genomics into NHS practice might be?
    a. Are there general ways these challenges be addressed? Prompts: *leadership, national guidance funding model, clinician time, support for clinicians to attend from clinical managers*

Setting up the prenatal ES service

1. Why do you think rapid prenatal ES was selected as one of the first tests to offer in the GMS?

2. Prior to the launch of the service had you been involved in discussions or planning for the overall prenatal ES service?

3. Do you know of any plans to audit or monitor the service so that you know implementation has been successful?

4. What do you think have been the main challenges of setting up a national prenatal ES service?
    a. Has anything been smoother than expected?
    b. What problems do you anticipate going forward
    c. Do you anticipate any differences in how individual GLH's will deliver the service?

5. What have been the main challenges of setting up the prenatal ES service at a local level?

6. What strategies have been used to raise awareness and offer training and education to professionals?
    a. Is any education and training of professionals being delivered nationally?
    b. Is any education and training occurring at a local level?

7. How prepared to offer ES are the fetal medicine professionals in your region?
    a. Are links between fetal medicine and genetics and the lab well established?
    b. How will genetics professionals support their fetal medicine colleagues as the service is established?

8. Can you see any barriers for the service locally?
   Prompt; engagement of local FMUs, cultural barriers in local populations

9. How have parents responded to being offered rapid ES?
    a. Do you have any thoughts about the information that has been developed for parents?
    b. What can we do to better support parents? Is there a need for more / different / other formats (online) information?
    c. The record of discussion is a new process for clinicians – how have you found using this as part of your practice?
    d. What do you think are the most important points to get across during pre-test counselling?
    Prompt: types of results such as no result or uncertain results / incidental findings / limitations of the technology?
    e. Do you anticipate any issues with equity of access in your region?
        a. Are there barriers to accessing ES for particular groups in your local community?
    f. Are there any strategies to address equity of access and inclusivity?

10. From your experience, what are some of the reasons that people decline prenatal exome sequencing?
    a. Are these the same or different to reasons to decline established prenatal genetic testing?

Thoughts on current practice guidelines

11. Are the national monthly meetings an effective way to discuss and effect changes in the service?
    a. Are there are there other benefits to these meetings?
    b. Are there disadvantages to these meetings?
    c. Other than these meetings are there ways of having your voice heard about the R21 service?

12. Do you have any thoughts on the approach of offering ES by referral through the genetics team?
    a. Looking to the future, do you think this test will continue to be offered by referral through the genetics team or will this ultimately shift to FMUs?

13. What do you think of the current eligibility criteria?
    a. Do you think the criteria will continue to change and evolve over time?

14. What do you think of the current approach to analysis where the scientists only look at certain genes by using a restricted panel?
    a. Do you think this will change over time?

15. What are your views on reporting only pathogenic and likely pathogenic findings? And only very limited reporting of VUS?

16. Is there anything you would like to change about the service as it develops?
    Prompts: review of eligibility criteria / routine process for re-analysis

17. Can you describe the practical aspects of organising these tests?

a. How is it decided who will be offered rapid exome sequencing?
b. Which professionals are involved in this process? (MDT meetings?)
c. How do MDT meetings work, could the MDT process be improved?
d. Have there been any issues in getting samples to the lab or communication with the lab?

18. What is the process for results interpretation and returning results?

a. How do the clinicians interact with the lab when results are returned? Do they support result interpretation?
b. Is there a standard process in place for decision making around variant classification?
c. How do MDT meetings work, could the MDT process be improved?

Other issues

19. Although this is not an option for the R21 service at present, do you think parents should have the choice to receive looked for additional findings?
    Prompt: benefits/ concerns/ what guidance should we follow – ACMG guidelines?

20. What do you think about offering ES as a non-invasive test?

21. Are there any ethical issues specific to prenatal ES that require consideration?
